# Fostering the clinician as teacher: A realist review

**DOI:** 10.1111/medu.15476

**Published:** 2024-07-21

**Authors:** Hiske Joanna Brouwer, Margot Barry, Manon Kluijtmans, Roger Anna Maria Joseph Damoiseaux, Esther de Groot

**Affiliations:** ^1^ Department of General Practice & Nursing Science, Julius Center for Health Sciences and Primary Care, University Medical Center Utrecht Utrecht University Utrecht The Netherlands; ^2^ Education Center, University Medical Center Utrecht Utrecht University Utrecht The Netherlands

## Abstract

**Background:**

Clinician‐teachers, physicians with educational responsibilities in either classroom or clinical setting, are assumed to add value by virtue of their dual role. The clinical responsibilities are often prioritised over the educational tasks. How and under which circumstances clinician‐teachers are able to perform their educational role and create added value for different stakeholders is currently unclear.

**Objectives:**

To identify for whom, how and under which circumstances educational activities executed by CTs by virtue of their dual role add value to others.

**Scope:**

CTs activities linking the system of education and clinical practice beyond direct patient interactions and purposefully executed.

**Methods:**

A realist review was conducted. Databases were searched in two stages: a narrow conventional search, followed by a comprehensive artificial intelligence‐aided search. Studies concerning clinician‐teachers' dual role were included. Realist analysis was applied to identify in which contexts resource mechanisms triggered reasoning mechanisms, which led to specific outcomes for different stakeholders.

**Results:**

Sixty‐six studies were included. In contexts where clinician‐teachers' dual role was formally recognised and valued, clinician‐teachers benefitted from the credibility and legitimacy bestowed on them, making the transfer of domain‐specific knowledge more impactful. In contexts where sociocultural differences between both systems existed, CTs were able to mediate and adjust recommendations aligned with stakeholders' perceived relevance. Also, contexts organised to support both roles made resource mechanisms more impactful. Clinician‐teachers added value to students' clinical competency and learning environment, and to educational organisations' curricular innovation. In their clinical workspace, clinician‐teachers added value by enhancing colleague physicians' teaching expertise, implementing educational innovations and recruiting students for scarce specialisms.

**Conclusion:**

Clinician‐teachers add value to students, colleague physicians and the clinical and educational contexts at large. Domain‐specific knowledge of both systems was important to gain credibility and achieve added value. Openness, formal recognition and allocated time for both roles in educational and clinical contexts towards the dual role are important to strengthen the impact of the dual role.

## Abstract 2

This realist review describes the added value stakeholders derive from individuals adopting the dual roles of clinician and teacher, highlighting means of maximizing their benefits.

## 1 INTRODUCTION

Physicians combining patient care with educational responsibilities in the classroom or in the clinical settings are often referred to as ‘clinician‐teachers’^1^, ^2^, ^3^ (CTs). They operate in two distinct socio‐cultural systems: education and clinical practice. It is noteworthy that even in the clinical setting these two systems remain distinct, despite their physical proximity. This review focuses on CTs who teach within classroom or clinical practice settings, both with and without formal teaching appointments.

Organisations invest in the employment of CTs, because it is assumed that these dual‐role professionals can effectively integrate the educational‐ and clinical systems and thereby add value to various stakeholders.^4^, ^5^ Integrating two systems requires CTs to balance priorities arising from settings which are subject to different priorities and different rules.^6^ In the clinical setting, e.g. patient care tasks can compete with educational tasks. The urgency and burden of responsibility associated with patient care often require them to be prioritised over educational activities; there is frequently insufficient training^7^ and time^8^, ^9^ for CTs' educational roles. CTs report the high workload associated with multiple roles,^8^, ^9^, ^10^, ^11^, ^12^, ^13^, ^14^ and experience difficulties in balancing priorities arising from two systems. Some even reported leaving the dual role to focus on one role only.^15^ It also appears that enthusing new graduates with dual roles that include educational tasks is not without challenge. Organisations experience substantial difficulties in the recruitment and retention of CTs,^16^, ^17^, ^18^, ^19^, ^20^, ^21^, ^22^, ^23^ to the extent that many organisations implemented tracks to encourage residents^24^, ^25^, ^26^, ^27^ and even undergraduate medical students^28^, ^29^, ^30^ to consider becoming CTs and to contribute to the educational system.

CTs consciously and explicitly aim to facilitate learning by employing educational activities that relate meaningfully to the clinical context. In these situations, a CTs' competence in both clinical and educational skills is required. It is the CT's ability to apply knowledge and skills from both the clinical and the educational contexts, that is said to provide rich learning opportunities for medical students. Literature shows that students learn by linking theory to practice more effectively and benefit from the CTs' educational activities, e,g. their explicit and deliberate role model function.^10^, ^31^, ^32^ Additionally, CTs report that they positively influence curriculum innovation processes as part of their educational role^2^, ^33^ and that their own clinical workplace improves given their educational responsibilities.^34^

Therefore, the added value of the CTs' educational role is known and uncontested. However, the contexts and mechanisms by which CTs are able to create added value through their educational role remain unclear. This poses a problem in structuring the dual role of CTs in a tenable manner. The lack of clarity creates difficulties in organising the dual role of CTs in order to overcome the challenges associated with the dual role as described above. How and under what conditions are CTs able to perform educational activities that add value, given that the educational role is often derogated to a lower priority? And since it is known from the literature that a dual role does not automatically result in the effective integration of two systems,^35^, ^36^ it is worth exploring how and under what conditions CTs are able to add value to different stakeholders by linking the educational‐ and clinical settings. Understanding the contexts and mechanisms might assist with the optimalisation of contexts to maximise the added value of CTs and counterbalance the disadvantages of the dual role.

The aim of this review is to describe the added value of CTs' educational activities executed by virtue of their dual role for students and other stakeholders, and how and under which circumstances this added value is achieved.

This review centres on CTs who teach within the classroom or clinical practice settings, both with and without formal teaching appointments. Box 1 outlines the reviews' scope. In particular, we focus on CTs' educational activities in situations where CTs metaphorically ‘take off their white coats’ and purposefully employ their dual role expertise. Therefore, we excluded those reporting undeliberate effects without a CT's effort, such as tacit role modelling, and those reporting CT teaching activities in close proximity to patients. Although we acknowledge the impact of situated learning, often occurring tacitly and incidentally in students during their clinical placements,^37^, ^38^ these situations lie beyond the scope of this review.

Box 1 **Scope of the review.**

**Characteristics of CTs:**

- Individuals that hold dual roles as both physician and educator;
- The educational responsibilities are either within classroom or within clinical practice settings. In the latter, we specifically look at educational moments outside of direct patient care delivery, and
- Educational responsibilities are executed both with and without formal teaching appointments.

**Characteristics of CTs' activities:**

- Educational activities that can only be executed by virtue of having a dual role;
- Educational activities that integrate the systems of education and clinical practice;
- Educational activities are executed purposefully, and
- Educational activities are executed without patient proximity or outside direct patient care delivery.

## 2 METHODS

We conducted a realist review following the Realist and Meta‐narrative Evidence Synthesis: Evolving Standards (RAMESES) for realist syntheses.^39^ This form of evidence synthesis gives an in‐depth understanding of a phenomenon by creating an explanatory theory about how (via which mechanisms), for whom and in what circumstances (contexts) outcomes are achieved or not.^40^

In the interest of feasibility, we restricted our search to physician‐teachers and did not include nursing‐ or allied health profession‐teachers.

### 2.1 Initial programme theory

As is central to realist approaches, we developed an initial programme theory (IPT) around the CTs dual role and their impact on stakeholders. The sources informing the IPT were: the researchers' prior knowledge of dual‐role professionals; preliminary data from the ‘narrow’ literature search (described below) and concepts purposefully selected from grand or middle‐range theories deemed to fit the IPT. These included boundary crossing theory,^41^ for we believed sociocultural differences between education and clinical practice would be spanned in some activities CTs execute to add value to their dual role. Also, informed by legitimacy theory^42^ we expected that CTs would be viewed as legitimate in the eyes of the stakeholders for being part of both systems. We remain open to being informed by the data with regard to other (learning) theories which might explain the outcomes of the educational role.

**FIGURE 1:**
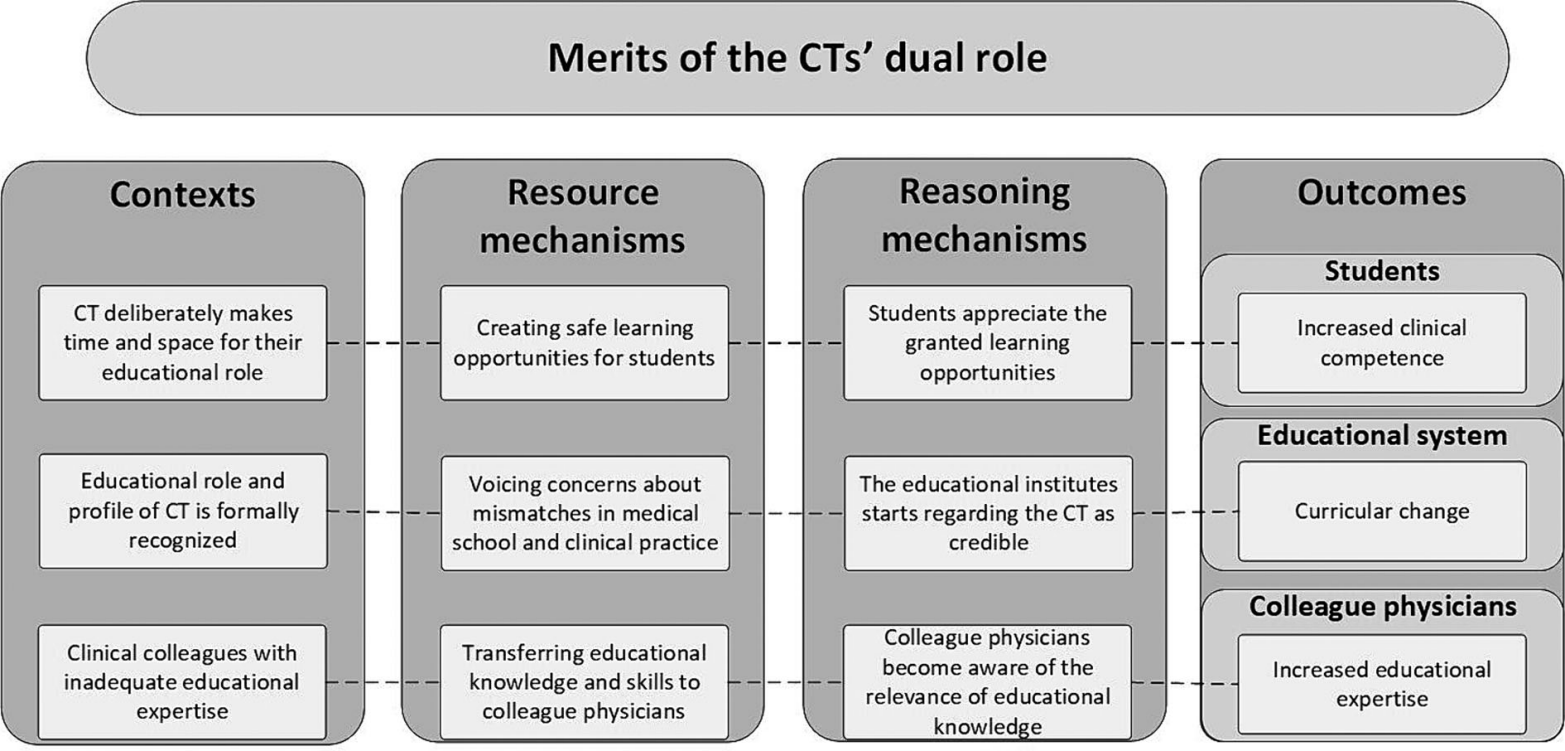
Initial programme theory.

In the IPT we assumed the existence and connections between contexts, mechanisms and outcomes, hence we represent their connection with dotted lines. Additionally, the IPT was created based on an adjusted CMO formula described by Dalkin et.al^43^. In this formula, mechanisms are subdivided into resource and reasoning: *resource* mechanisms are components of an intervention, in our case activities in which the CT uses clinical expertise in the education system or didactic expertise in the clinical system, that are introduced into a certain context; r*easoning* mechanisms describe the ways in which this activity changes the reasoning of stakeholders in a manner that contributes to achieving outcomes. Resource mechanisms are coupled to the reasoning mechanism in each CMO. Figure 1 depicts the IPT.

### 2.2 Search process and appraisal

A three‐stage search strategy was used. First, we conducted a narrow conventional search on 1 January 2021, in the databases Scopus, EmBase, PubMed and ERIC, using synonyms of ‘clinician‐teacher’, ‘dual role’ and ‘integrating clinical practice and education’ (see appendix A). For identifying relevant terms for ‘integrating clinical practice and education’, we drew inspiration from boundary‐spanning literature. The database search was not limited by date and included English‐language texts only. We included studies that reported the enactment, outcomes, barriers or facilitators relating to CT activities that integrate education and clinical practice. Whilst we included empirical studies only, the rigour of the research method was not examined as an inclusion criterium. The relevance and richness of the content was considered more important. Three authors (HB, EdG, MB) screened abstracts and subsequent full texts independently of each other. Conflicts were resolved through discussions. When in doubt the other authors were consulted. Secondly, on 1 April 2021, we performed a second wider, more comprehensive search using the artificial intelligence programme ASReview^44^ to aid the screening process with a broader search string than in the conventional search (see Appendix A). ASReview uses machine‐ and active learning based on prior decisions to continually rearrange imported records, in order to present those articles which it assesses as ‘most likely to be relevant’ first. We used the default model (Naïve Bayes, TF‐IDF, Max). Eleven of the included and eleven of the excluded articles from the conventional search served as prior knowledge to train ASReview. We continued screening until our pre‐determined stopping rule of 80 non‐relevant abstracts was reached. The results of the AI‐based selection were appraised using the same criteria as for the initial narrow search. Thirdly, forward‐backward reference screening was done in September 2023.

### 2.3 Data analysis

Characteristics of included articles were initially extracted into Microsoft Word to obtain familiarity with the data (HB, MB). Next, we coded full‐text articles using NVivo version 12 software (HB). We first coded for resource mechanisms (CTs activities by virtue of their dual role) and outcomes. Then, we identified repeated patterns (demi‐regularities) between certain contexts and outcomes. Lastly, we searched and inferred explanations (reasoning mechanisms) as to how these contexts resulted in the outcomes. Emergent findings were regularly discussed at research team meetings, to ensure coherence and credibility of the explanations. Extracted data was confirmed, refined and refuted, which eventually constructed the final programme theory.

During the analysis process, it became apparent that certain sources for constructing the CMOs originated from the stakeholders themselves (i.e., personally experienced), while others stemmed from the CT (i.e., projected experience). We will elucidate this distinction in the presentation of the results.

### 2.4 Reflexivity

All members of the research team have working experience and theoretical knowledge of the added value of dual roles; HB, EdG and RD work within a GP training institute, RD is a practicing GP and head of a GP training institute, whilst MB and MK are researchers focusing on dual roles in the education‐research‐practice triangle. During all stages of the research, the researchers challenged each other's expectations and assumptions to remain open to being informed by the data.^45^ We acknowledge the dialogue in the current literature on the interpretation of data, for instance about labelling contexts and mechanisms^43^ and are aware that information we labelled as contexts, others may perceive as mechanisms and vice versa.

## 3 RESULTS

**FIGURE 2:**
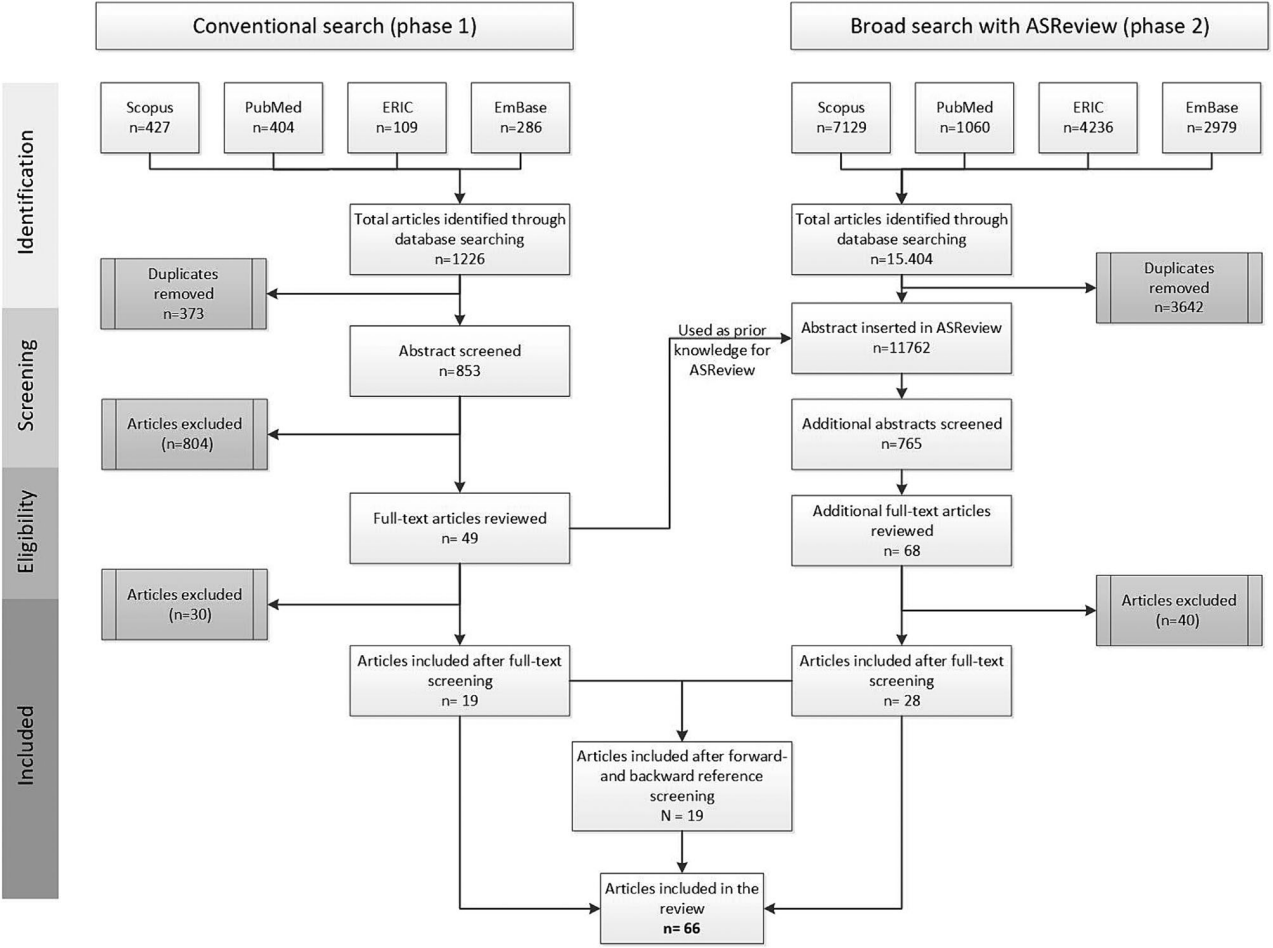
Flowchart of in‐ and excluded articles.

Of the 853 unique articles found using the conventional search, 19 were included (see Figure 2). Via the broader search 11.762 unique articles were found and sorted on relevance. After screening the first 765 of the articles our stopping rule was reached. Screening of these 765 articles led to 28 additional included articles. Finally, using forward‐ and backward reference searching another 19 articles were included, resulting in a total of 66 articles used in this review. Appendix B presents a reference list of the included articles.

Included articles represented four regions categorised by the World Health Organisation^46^: the Americas (n = 33); Europe (n = 22); Western Pacific (n = 10); Africa (n = 1); South‐East Asia (n = 0) and Eastern Mediterranean region (n = 0) whereby the global north was represented more comprehensively (n = 64) than the global south (n = 2). Research methods were qualitative (n = 59), mixed methods (n = 4) and quantitative (n = 3). Most research concentrated on the CT as the subject of the study (n = 54), and some included stakeholders as subjects: students (n = 20), clinical colleagues (n = 5) and patients (n = 2). Most articles described CTs educational system situated in the clinical setting (n = 59), others in the classroom setting (n = 3) or both (n = 4). There is considerable variation in the manner in which CT roles are implemented internationally.

### 3.1 Adjusted programme theory

**FIGURE 3:**
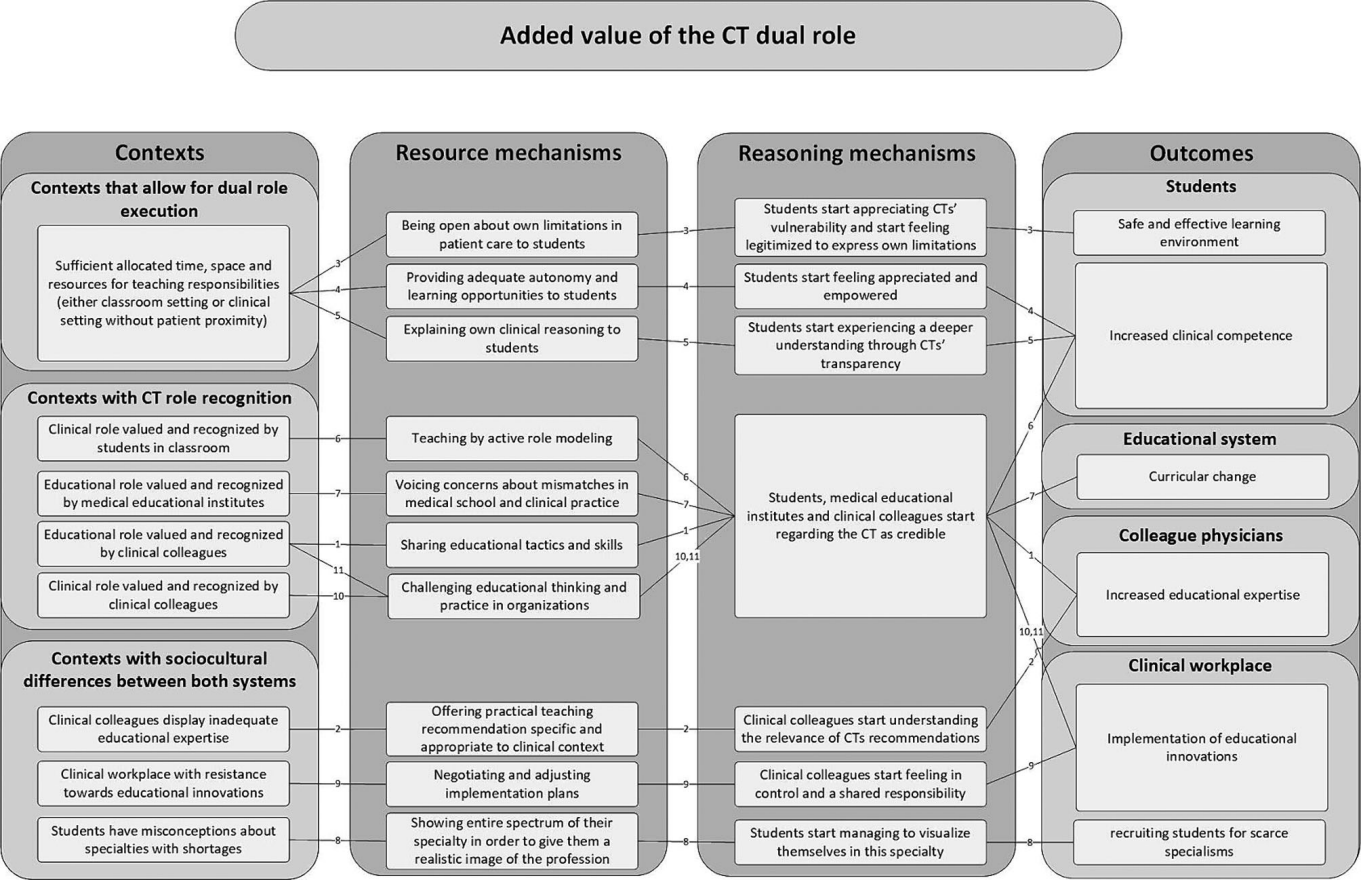
Adjusted programme theory. CT: clinician‐teacher. Numbers in lines correspond with CMO numbers in Table 1.

Subsequent to the analysis of the entire data set, we conceptualised an adjusted programme theory (APT) (see Figure 3), with consistencies and adjustments with respect to the IPT.

The main consistency was the use of the alternative CMO‐formula which was carried forward to the APT as it represented the data of included studies well.

The main adjustments included a refinement of our idea about the CMOs with additional contexts, mechanisms and outcomes. The APT is more detailed with confirmed connections (solid lines) between context, mechanisms and outcomes found in the data. Important resource mechanisms added to the APT include a range of educational skills needed on the part of the CT to trigger reasoning mechanisms. These skills include conscious active role modelling, the ability to share own clinical reasoning at an appropriate level with students and maintaining an openness about own limitations.

Furthermore, the APT elucidates the value of engaging with sociocultural differences between the clinical and educational contexts constructively rather than avoiding or ignoring these differences. In addition to boundary crossing, we also saw that cognitive learning theories are helpful in understanding certain mechanisms.

The data analysis resulted in eleven unique CMOs. The eleven CMOs are described in Table 1 and arranged per benefitting stakeholder or system. The table also provides information on whether CMOs are based on personal‐ and/or projected experiences.

**TABLE 1:** All CMO configurations per stakeholder group.

Nb, refs	Context	Resource mechanism	Reasoning mechanism	Outcome	Personal/projected experience*
Colleague physicians in the clinical setting					
CMO1^34^, ^47^, ^48^, ^49^	CT's educational role and profile is formally recognised and supported by clinical colleagues and management	CT shared educational tactics and skills with colleague physicians	Colleague physicians ascribed a high level of credibility to the information given	Improved teaching competency	4/0
CMO2^34^, ^47^	Colleague physicians (with less educational experience than the CT) display inadequate educational expertise when working with students or colleagues	CT offered relevant practical teaching recommendations, specific and appropriate to the clinical context	Colleague physicians understand the relevance and a positive disposition towards the recommendations		0/2
Students in the clinical and classroom setting					
CMO3^31^, ^50^, ^51^, ^52^, ^53^, ^54^, ^55^, ^56^, ^57^, ^58^, ^59^, ^60^, ^61^, ^62^, ^63^, ^64^	The educational system, either within clinical or classroom setting, with sufficient allocated time and equipped space for CTs educational responsibilities.	CT admitted his own limitations and uncertainties with regard to patient care to the students	Students appreciated the transparency and felt legitimised to express their limitations as well	Safe and stimulating learning environment	5/11
CMO4^25^, ^31^, ^47^, ^50^, ^51^, ^54^, ^56^, ^62^, ^65^, ^66^, ^67^, ^68^, ^69^, ^70^, ^71^, ^72^, ^73^, ^74^, ^75^, ^76^, ^77^, ^78^, ^79^, ^80^, ^81^, ^82^, ^83^		CT provided students with learning opportunities and autonomy appropriate to students' level of competence, given familiar clinical challenges	Students appreciated and felt empowered by the well‐aligned tasks	Enhancement of students' clinical competency	12/15
CMO5^31^, ^50^, ^52^, ^55^, ^59^, ^70^, ^74^, ^75^, ^77^, ^84^, ^85^, ^86^, ^87^, ^88^, ^89^, ^90^		CT explicitly explained the clinical reasoning behind his actions to the students	Transparency fostered a deeper understanding of the observed in students		7/9
CMO6^18^, ^91^, ^92^	CTs' clinical role is recognised and valued by students in the classroom	CT taught students by means of an active role model function and thereby showed they are still actively working as a physician	Students ascribed a high level of credibility and legitimacy to the information and believe the information is useful		2/1
The educational system					
CMO7^17^, ^49^, ^61^, ^75^, ^86^, ^91^, ^92^, ^93^, ^94^, ^95^, ^96^, ^97^	CTs' educational role is recognised and valued by faculty members in the medical institute	CT voiced concerns about the mismatches between clinical practice and the curriculum	Faculty members valued the information, as this feedback came from clinical practice	Curriculum changes	0/5
The clinical workplace					
CMO8^17^, ^73^, ^75^, ^76^, ^86^, ^98^, ^99^, ^100^, ^101^, ^102^	Students have unfamiliarity or misconceptions concerning a specialty with shortages of physicians	CT took time and effort to show students the entire spectrum of their specialty in order to give them a realistic image of the profession, including work‐life balance	Students managed to visualise themselves in this specialty	Students display more interest in the specialty with shortages and considered it as a future career choice	5/5
CMO9^58^, ^103^, ^104^, ^105^, ^106^	Clinical team displays resistance to educational innovation	CT negotiated goals and adjusted plans for the implementation of educational innovations	Clinician team felt in control and a shared responsibility	Implementation of educational innovations	2/3
CMO10^96^, ^103^, ^104^, ^105^, ^106^	CTs' clinical role is valued being part of the clinical team	CT had a mandate to contribute suggestions and advice towards educational innovation	Clinical team perceived suggestions as trustworthy		1/4
CMO11^47^, ^49^, ^58^, ^103^, ^104^, ^105^, ^106^, ^107^	CT educational role and profile is formally recognised and supported by colleagues and management	CT challenged educational thinking and practices in the organisation through the use of relevant and innovative evidence	Clinical team ascribed a high level of credibility to the information		2/6

* Outcomes described by members of the stakeholder group are reported as personal experiences; outcomes described by the CT were reported as projected experiences.

Outcomes were found on an individual and organisational level. CTs added value in creating a safe learning environment for students in both the clinical and classroom settings. CTs added value by facilitating students' acquisition of clinical skills. CTs added value through colleague physicians' educational expertise. On an organisational level, CTs added value to educational organisations through curricular innovation, and to the clinical workplace through the implementation of educational innovations and the recruitment of students for scarce specialisms.

Several contexts were inferred that triggered or facilitated CTs to employ their dual role through activities that integrated the systems of education and clinical practice. In the eleven CMOs, three main, overarching contexts could be inferred: ‘contexts with sociocultural differences between the educational and clinical system’, ‘contexts with formal recognition and value towards the CTs’ roles' and ‘contexts organised to support both roles’.

### 3.2 Added value of CTs dual role in contexts with role recognition

Formally recognising and valuing the CTs' educational role appeared important in achieving added value of the CTs' dual role. In contexts where role recognition was present, the CT was able to transfer knowledge from one system to the other. For instance, by voicing concerns about mismatches in medical school and clinical practice or by sharing educational tactics and skills with colleague physicians. Transfer of knowledge in contexts where the CT role was valued, triggered reasoning mechanisms in stakeholders: they perceived clinician‐teachers as credible, trustworthy and legitimate. Below, we describe two CMOs in this category, accompanied by information on its construction.

CMO1 (in Table 1). In a context where the CTs educational role and profile are formally recognised and supported by colleagues and management (context), the CT shared educational tactics and skills with colleague physicians (resource mechanism), who ascribed a high level of credibility to this information (reasoning mechanism) and hence implemented educational tactics in their own teaching with students (outcome).

This CMO was constructed by both positive^47^, ^48^ and negative^34^, ^49^ examples whereby in the latter, the absence of a supportive context did not trigger the sharing of educational tactics and whereby the CT did not enjoy credibility to be able to advise colleagues on education.

CMO7 (in Table 1): In a context where the CT is viewed as a competent and credible teacher by faculty members in the medical school (context), the CT voiced concerns about the mismatches between clinical practice and the curriculum (resource mechanism), which faculty members valued as this feedback came from clinical practice (reasoning mechanism), therefore resulting in curriculum changes (outcome).

Some sources described positive examples,^75^, ^91^ but we found mostly negative examples. CTs reported mismatches and concerns which were never shared due to feelings of powerlessness in contexts without CT role appreciation.^17^, ^94^, ^95^ Or, in situations where concerns were shared, feedback was not acted upon by the education institute.^93^, ^96^, ^97^ In some instances CTs made changes in their own educational practices (microlevel) to circumvent issues with the curriculum which they perceived as mismatch with their clinical practice.^61^, ^86^, ^94^, ^96^

Findings in this category could fit source credibility theory^108^ and legitimacy theory,^42^ which state that the credibility of communicated information is heavily influenced by the perceived credibility of the communicator.

### 3.3 Added value of CTs dual role in contexts with sociocultural differences between the systems of education and clinical practice

In this category, sociocultural differences between the system of education and clinical practice triggered CTs to utilise their dual role in bridging the gap between both systems. Sociocultural differences existed for instance in clinical workplaces with resistance to educational innovations, as exhibited by the persistence of outdated or impractical educational systems without willingness to change. Or, in situations where discrepancies existed between students' perceptions of a particular medical specialty and the actual realities inherent to that specialty. Interestingly, these types of contexts triggered CTs to use their domain‐specific knowledge on both systems and adjust or customise knowledge transfer, so that it suited both system demands. For instance, by offering teaching recommendations specific and appropriate to the clinical context and when negotiating and adjusting implementation plans to suit them to the demands of the clinical workplace. These activities triggered reasoning mechanisms in stakeholders such as feeling in control, feeling a shared responsibility and understanding the relevance.

Below, we describe one exemplary CMO.

CMO9 (in Table 1). In a context where the clinical team displays resistance to educational innovation (context), the CT negotiated goals and adjusted plans for the implementation of educational innovations (resource mechanism) in a manner that resulted in feelings of being in control and shared responsibility on the part of the clinical team (reasoning mechanism) leading to the implementation of educational changes. (outcome).

Findings in this category could fit the theory of boundary spanning,^41^ in which activities by brokers are being used to bridge differences between settings through interacting and translating concepts across them.

### 3.4 Added value of CTs dual role in contexts that supported the execution of both roles

In order to achieve many student outcomes, sources described the importance of contexts that supported the execution of both CT roles. In many situations, time constraints forced CTs to prioritise clinical work over teaching responsibilities, thereby restricting teaching activities and subsequent student outcomes. When both roles were well organised, for instance when CTs had allocated time for their educational tasks either within the clinical setting or classroom setting, or when clinical colleagues were able to take over CTs' clinical tasks, CTs were better able to perform activities that integrated their clinical experience in education. For instance, by being open to their own limitations in patient care, by explaining their clinical reasoning behind actions and by well‐aligning autonomy and learning opportunities with students' level of competency given familiar clinical challenges in their own clinical practice. By integrating the system of clinical practice into their teaching, reasoning mechanisms such as appreciation and empowerment were triggered in students, resulting in safe and stimulating learning environments and the enhancement of students' clinical competence. The classroom setting was rarely described as a context in the data.^57^, ^64^, ^68^, ^76^, ^87^

Below, an exemplary CMO is described, along with information on its construction.

CMO4 (in Table 1). In a context where the CT has allocated time for teaching responsibilities, either within classroom or clinical setting (context) the CT provided students with learning opportunities and autonomy appropriate to the students' level of competence (resource mechanism), making the students appreciate and feel empowered by the well‐aligned tasks (reasoning mechanism), resulting in the growth of the students' clinical competence (outcome).

We found multiple sources describing the non‐achievement of this outcome when CTs were unable to find the time to get to know the student and align learning opportunities with the students' level of competence: expressions of restriction when unchallenging tasks were given, and discomfort when too much autonomy was granted.^54^, ^56^, ^67^, ^68^, ^69^, ^73^, ^109^

## 4 DISCUSSION

Our review set out to answer the questions for whom, how and in what circumstances CTs are able to add value to their stakeholders by virtue of their dual role through integrating activities linking the clinical‐ and educational settings. We found that important facilitating contexts by which CTs were able to utilise their dual role included recognition and value towards the role and sufficient allocated time and space to execute both roles. The credibility, trustworthiness and legitimacy bestowed upon the CT are important mechanisms in their ability to add value. CTs added value to persons (colleagues and students) and systems with (in) which they work (the clinical workplace and educational system). Below, we discuss the main messages.

Firstly, discernible differences in the actions undertaken by CTs existed, that appeared to depend on the extent to which their role was appreciated or met with resistance. When CTs role and profile were valued and recognised, they could integrate the knowledge of the other system, and stakeholders accepted this integration due to the perceived credibility they bestowed upon CT. Conversely, in instances where resistance was encountered, CTs had to adapt and align the information from the alternative system so that stakeholders were able to perceive the relevance of the information. A conscious effort was required to plan, set educational goals and scaffold student boundary crossing too. The CTs didactic responsibility went beyond imparting theoretical knowledge. It included voicing and discussing the observed differences between the two systems (clinical and educational) for the benefit of stakeholder reflection and learning. The CTs efforts that address the challenges of working across and in between the two activity systems are frequently not pertinently visible to stakeholders yet require time and expertise from the CT. The importance of valuing the CTs educational and clinical role for creating impact could not only contribute to their ability to deliver results but also influence the CTs inclination to remain in the dual role. Feeling valued contributed to impactful work^110^, ^111^ and could assist in the retention of CTs.^112^, ^113^, ^114^, ^115^, ^116^ We highlight the importance for educational institutions and clinical workplaces to create atmospheres in which the CTs dual role is valued thus facilitating the positive effects of integrating both systems. This could, e.g. be done by explicitly asking for their input in curriculum development, which we infrequently observed in our data.

Furthermore, the credibility and legitimacy attributed to CTs underline the advantage of physician‐educators over non‐physician medical educators (NPME). This aligns with research on ingroup‐outgroup dynamics, emphasising how disciplinary cultures bestow legitimacy and authority within their respective fields.^117^ NPMEs may face scepticism because of perceived differences in culture, teaching approaches and their ability to relate to clinical realities.^117^ Publications describing NPME teaching experiences indeed revealed challenges in understanding medical jargon and commonalities^92^ and that students needed time to recognise them as educators.^118^ Additionally, NPMEs were less able to estimate a learner's level of knowledge,^118^ which our results showed as an important mechanism contributing to student learning.

Lastly, CTs having sufficient allocated time and space for the execution of both roles, was an important context for achieving student outcomes. Time pressures, prioritising clinical tasks above educational tasks and the importance of protected time for the educational role have been described in the literature.^119^, ^120^ These factors may contribute to the limited number of CMOs describing CTs participation in outcomes related to the educational system. It remains challenging to balance priorities arising from two different systems.

### 4.1 Methodological strengths and challenges

A strength of this review is the realist approach, in which contexts were revealed with a larger or lesser effect of CTs' dual role in others, thereby making the transfer of our results practical.^121^, ^122^ Another strength was the use of ASReview that enabled us to screen and include a large set of additional literature, which made our review more rigorous, while at the same time feasible. Another strength is the elucidation of stakeholder experiences, as opposed to only CTs perceptions of outcomes, especially considering the limited number of personal experiences reported in the literature. Lastly, although the forward‐backward search revealed many additional articles, no new CMO configurations could be created using these articles, implying data saturation.

A limitation of this study is the exclusion of articles concerning CTs *implicit* BS actions. Therefore, the impact of unconscious BS activities, like the formation of students' professional identity when observing implicit role‐modelling of CTs,^123^, ^124^, ^125^ is not described. Another limitation is the conscious decision to limit the search to the medical profession for feasibility reasons, whilst many other healthcare professions also employ CTs. We acknowledge the fact that most included studies were conducted within Western countries.

### 4.2 Suggestions for future research

There seemed to be less research published on CTs working in the global south and in the interest of equity, diversity and inclusion, we emphasise the importance of acknowledging and addressing potential structural barriers that researchers in the global south may face when attempting to contribute to publications on this topic. There was a distinction observed among sources conceptualising learning as sociocultural phenomenon or as a cognitive activity. We believe studies exploring this subject within the perspective of sociocultural learning gave a richer view, therefore we suggest future research in this perspective.

To add to current insights, we recommend that future research integrate more stakeholder experiences to glean insights into the authentic impact on others. These personal experiences were especially limited when investigating effects on educational institutes and the clinical workplace. In addition to studies aimed at better understanding CT's value, intervention studies optimising contexts to make added values of CTs more impactful would contribute to the literature. Lastly, although a substantial part of medical education takes place within classroom setting, remarkably few publications regarding this topic in classroom setting were found. CTs teaching in classroom settings consciously chose a CT career, creating a larger call for research within this context.

## 5 CONCLUSION

This realist review confirms that CT's educational activities executed by virtue of their dual role result in added value for colleague physicians and students as well as the clinical‐ and educational settings at large. The review adds new knowledge on how this added value may be fostered: important mechanisms in achieving added value were the credibility, trustworthiness and legitimacy bestowed upon the CT. Insights of this study allow for the optimalisation of contexts to maximise the added value of CTs and counterbalance the disadvantages of a dual role. Openness and appreciation of the educational role of CTs by both the educational and clinical systems are important to strengthen the impact of CT's dual role. In educational institutes, this could be achieved by explicitly asking for CT's input in curriculum development. In the clinical workplace, this could be achieved by management support and formally recognising the dual role, for instance by providing time and opportunities for their educational role.

## AUTHOR CONTRIBUTIONS

HB, EdG, MK and RB contributed to the conception and design of the review, the search strategy was created by HB and EdG, articles were acquired by HB and the selection of articles was done by HB, EdG and MB. Coding and analysis were initially done by HB and MB and all authors were consulted and substantially contributed to the analysis and interpretation of data. HB and MB wrote the first draft of the manuscript, which was then critically revised by EdG, MK and RD. All authors approved the final manuscript for publication and agreed to be accountable for all aspects of the work.

## CONFLICT OF INTEREST STATEMENT

None.

## ETHICS STATEMENT

Not applicable.

## Supporting information


**Appendix A.** Search strings

**Appendix B.** List of all 66 included articles^1^, ^2^, ^3^, ^4^, ^5^, ^6^, ^7^, ^8^, ^9^, ^10^, ^11^, ^12^, ^13^, ^14^, ^15^, ^16^, ^17^, ^18^, ^19^, ^20^, ^21^, ^22^, ^23^, ^24^, ^25^, ^26^, ^27^, ^28^, ^29^, ^30^, ^31^, ^32^, ^33^, ^34^, ^35^, ^36^, ^37^, ^38^, ^39^, ^40^, ^41^, ^42^, ^43^, ^44^, ^45^, ^46^, ^47^, ^48^, ^49^, ^50^, ^51^, ^52^, ^53^, ^54^, ^55^, ^56^, ^57^, ^58^, ^59^, ^60^, ^61^, ^62^, ^63^, ^64^, ^65^, ^66^

**Appendix C.** Supportive quotes for CMO constructions

## Data Availability

The data that support the findings of this study are available from the corresponding author upon reasonable request.
