# Supplementary material for: Fostering the clinician as teacher: A realist review
Source: Med Educ. 2024 Jul 21;59(2):151–63. doi: 10.1111/medu.15476 (PMC11708814; doi:10.1111/medu.15476)
Supplement: Supplementary file 1 — Appendix A. Search strings [file MEDU-59-151-s001.docx]

**Appendix A – search strings**

Figure 1 depicts the narrow search, figure 2 depicts the comprehensive search

**Figure 1: narrow search**

| **Databases** | **Synonyms of “clinician-teacher”, “dual role” and “integrating clinical practice and education”** |
| --- | --- |
| Scopus | ( TITLE-ABS-KEY ( "clinician teacher*" OR "clinician educator*" OR "clinical teacher*" OR "clinical educator*" OR "practitioner teacher*" ) OR ( TITLE-ABS-KEY ( clinician* OR physician* OR doctor* OR "medical practitioner*" ) AND TITLE-ABS-KEY ( teach OR teaches OR teaching OR teacher* OR educate* OR educating OR educator* OR instruct* OR educationalist* OR lecturer* ) AND TITLE-ABS-KEY ( "dual role*" OR "hybrid role*" OR "double role*" OR "dual function*" OR "double function*" OR "hybrid function*" OR "dual profession*" OR "hybrid profession*" OR "double profession*" OR ( two PRE/2 roles ) OR ( two PRE/2 functions ) OR ( two PRE/2 professions ) OR "blended professional*" ) ) ) AND ( TITLE-ABS-KEY ( broker* OR ( boundar* W/5 spanner* ) OR ( boundar* W/5 crosser* ) OR gatekeeper* OR brokering OR ( boundar* W/5 crossing ) OR ( boundar* W/5 spanning ) OR ( ( ( cross OR across OR between ) W/5 boundar* ) AND ( learn* OR collaborat* ) ) OR ( boundar* AND ( ( cross OR across OR between OR different ) W/5 ( practices OR organizations OR settings OR workplaces OR institutes OR agencies ) ) ) OR "boundary role*" ) OR TITLE-ABS-KEY ( liaison* OR "go between*" OR intermediator* OR navigator* OR "linkage agent*" OR "boundary worker*" OR ( ( linking OR connecting OR integrating ) W/10 boundaries ) OR "role model*" OR "disseminated learning" ) OR ( TITLE-ABS-KEY ( building W/10 bridge* ) AND SUBJAREA ( medi OR nurs OR vete OR dent OR heal OR mult ) ) OR TITLE-ABS-KEY ( socialization OR "informal learning" OR "non-formal learning" OR translator* OR facilitator* OR ( ( diffuse* OR transfer* OR connect* OR link* OR exchang* OR disseminat* OR shar* OR facilitat* ) W/3 ( knowledge OR information ) ) OR ( ( link* OR transfer* OR connect* OR work OR working OR exchang* OR communicat* ) W/5 ( practices OR organizations OR settings OR workplaces OR institutes OR agencies ) ) OR ( hybridization W/5 "institutional logics" ) OR "organizational barrier*" ) ) |
| Pubmed | (( (((("clinician educator*"[Title/Abstract]) OR ("clinical teacher*"[Title/Abstract])) OR ("clinical educator*"[Title/Abstract])) OR ("practitioner-teacher*"[Title/Abstract])) OR ("clinician teacher*"[Title/Abstract]) ) OR (( (((Clinician*[Title/Abstract]) OR (Physician*[Title/Abstract])) OR (Doctor*[Title/Abstract])) OR (“Medical practitioner*”[Title/Abstract]) AND ((((((((((teach[Title/Abstract]) OR (teaches[Title/Abstract])) OR (teaching[Title/Abstract])) OR (teacher*[Title/Abstract])) OR (educate[Title/Abstract])) OR (educates[Title/Abstract])) OR (educating[Title/Abstract])) OR (educator*[Title/Abstract])) OR (instruct*[Title/Abstract])) OR (educationalist*[Title/Abstract])) OR (lecturer*[Title/Abstract]) ) AND ( (((((((((((("Dual role*"[Title/Abstract]) OR ("Hybrid role*"[Title/Abstract])) OR ("Double role*"[Title/Abstract])) OR ("Dual function*"[Title/Abstract])) OR ("Hybrid function*"[Title/Abstract])) OR ("Double function*"[Title/Abstract])) OR ("Dual profession*"[Title/Abstract])) OR ("Hybrid profession*"[Title/Abstract])) OR ("Double profession*"[Title/Abstract])) OR ("Two roles"[Title/Abstract])) OR ("Two functions"[Title/Abstract])) OR ("Two professions"[Title/Abstract])) OR ("blended professional*"[Title/Abstract]) ))) AND ( ( (((((((Broker*[Title/Abstract]) OR (boundary spanner[Title/Abstract])) OR (boundary crosser[Title/Abstract])) OR (gatekeeper*[Title/Abstract])) OR (brokering[Title/Abstract])) OR (boundary crossing[Title/Abstract])) OR (boundary spanning[Title/Abstract])) OR ("boundary role*"[Title/Abstract]) OR ((learn*[Title/Abstract]) OR (collaboration*[Title/Abstract]) AND (("cross boundar*"[Title/Abstract]) OR ("across boundar*"[Title/Abstract])) OR ("between boundar*"[Title/Abstract])) OR (((((practices[Title/Abstract]) OR (organisations[Title/Abstract])) OR (settings[Title/Abstract])) OR (workplaces[Title/Abstract])) OR (institutes[Title/Abstract])) OR (agencies[Title/Abstract]) AND (((across[Title/Abstract]) OR (cross[Title/Abstract])) OR (between[Title/Abstract])) OR (different[Title/Abstract]) AND (boundar*[Title/Abstract]) ) OR ( (((((((Liaison*[Title/Abstract]) OR ("Go between*"[Title/Abstract])) OR (Navigator*[Title/Abstract])) OR (Intermediator*[Title/Abstract])) OR ("Linkage agent*"[Title/Abstract])) OR ("Boundary worker*"[Title/Abstract])) OR ("Role-model*"[Title/Abstract])) OR ("Disseminated learning"[Title/Abstract]) OR ((((linking[Title/Abstract]) OR (connecting[Title/Abstract])) OR (integrating[Title/Abstract])) AND (boundaries[Title/Abstract])) OR ((building[Title/Abstract]) AND (bridge*[Title/Abstract])) ) OR ( ((((((Socialization[Title/Abstract]) OR ("Informal learning"[Title/Abstract])) OR ("Non-formal learning"[Title/Abstract])) OR (Translator*[Title/Abstract])) OR (Facilitator*[Title/Abstract])) OR ("Organizational barriers"[Title/Abstract])) OR (((knowledge[Title/Abstract]) OR (information[Title/Abstract])) AND ((((((((diffus*[Title/Abstract]) OR (transfer*[Title/Abstract])) OR (connect*[Title/Abstract])) OR (link*[Title/Abstract])) OR (exchang*[Title/Abstract])) OR (shar*[Title/Abstract])) OR (disseminat*[Title/Abstract])) OR (facilitate*[Title/Abstract]))) OR (((((((practices[Title/Abstract]) OR (organizations[Title/Abstract])) OR (settings[Title/Abstract])) OR (workplaces[Title/Abstract])) OR (institutes[Title/Abstract])) OR (agencies[Title/Abstract])) AND (((((((link*[Title/Abstract]) OR (transfer*[Title/Abstract])) OR (connect*[Title/Abstract])) OR (work[Title/Abstract])) OR (working[Title/Abstract])) OR (exchang*[Title/Abstract])) OR (communicat*[Title/Abstract]))) ) ) |
| EmBase | (( 'clinician-teacher*':ti,ab,kw OR 'clinician-educator*':ti,ab,kw OR 'clinical-teacher*':ti,ab,kw OR 'clinical-educator*':ti,ab,kw ) OR (( (clinician:ti,ab,kw OR physician:ti,ab,kw OR doctor*:ti,ab,kw OR 'medical practitioner*':ti,ab,kw) AND (teaching:ti,ab,kw OR teach:ti,ab,kw OR teaches:ti,ab,kw OR teacher:ti,ab,kw OR teachers:ti,ab,kw OR educate:ti,ab,kw OR educates:ti,ab,kw OR educating:ti,ab,kw OR educator*:ti,ab,kw OR instruct*:ti,ab,kw OR educationalist*:ti,ab,kw OR lecturer*:ti,ab,kw) ) AND ( 'dual role*':ti,ab,kw OR 'hybrid role*':ti,ab,kw OR 'double role*':ti,ab,kw OR 'dual function*':ti,ab,kw OR 'hybrid function*':ti,ab,kw OR 'double function*':ti,ab,kw OR 'dual profession*':ti,ab,kw OR 'hybrid profession*':ti,ab,kw OR 'double profession*':ti,ab,kw OR ((two NEXT/2 functions):ti,ab,kw) OR ((two NEXT/2 roles):ti,ab,kw) OR ((two NEXT/2 professions):ti,ab,kw) OR 'blended professional*':ti,ab,kw ))) AND (( broker*:ti,ab,kw OR ((boundary NEAR/5 spanner*):ti,ab,kw) OR ((boundary NEAR/5 crosser*):ti,ab,kw) OR ((boundary NEAR/5 spanning):ti,ab,kw) OR ((boundary NEAR/5 crossing):ti,ab,kw) OR gatekeeper*:ti,ab,kw OR brokering:ti,ab,kw OR 'boundary role':ti,ab,kw OR ((((cross OR across OR between) NEAR/5 boundar*):ti,ab,kw) AND (learn*:ti,ab,kw OR collaboration*:ti,ab,kw)) OR (boundar*:ti,ab,kw AND (((across OR cross OR between OR different) NEAR/5 (practices OR organisations OR settings OR workplaces OR institutes OR agencies)):ti,ab,kw)) ) OR ( liaison*:ti,ab,kw OR 'go between*':ti,ab,kw OR intermediator*:ti,ab,kw OR navigator*:ti,ab,kw OR 'linkage agent*':ti,ab,kw OR 'boundary worker*':ti,ab,kw OR (((linking OR connecting OR integrating) NEAR/10 boundaries):ti,ab,kw) OR ((building NEAR/10 bridge*):ti,ab,kw) OR 'role-model*':ti,ab,kw OR 'disseminated learning':ti,ab,kw ) OR ( socialization:ti,ab,kw OR 'informal learning':ti,ab,kw OR 'non-formal learning':ti,ab,kw OR translator*:ti,ab,kw OR facilitator*:ti,ab,kw OR ((hybridisation NEAR/5 'institutional logics'):ti,ab,kw) OR 'organizational barriers':ti,ab,kw OR (((diffus* OR transfer* OR connect* OR link* OR exchange* OR exchang* OR disseminat* OR shar* OR facilitate*) NEAR/3 (knowledge OR information)):ti,ab,kw) OR (((link* OR transfer* OR connect* OR work OR working OR exchang* OR communicat*) NEAR/5 (practices OR organizations OR settings OR workplaces OR institutes OR agencies)):ti,ab,kw) )) |
| ERIC | ( (‘clinician educator’ OR ‘clinical educator’ OR ‘clinician teacher’ OR ‘clinical teacher’) OR (Clinician OR physician OR doctor OR ‘medical practitioner’) AND (teach OR educat OR instructor OR lecturer) AND ( (dual OR hybrid OR double OR two ) AND (role OR function OR profession) ) ) AND (Broker OR ‘boundary span*’ OR ‘boundary cros*’ OR gatekeeper OR brokering OR ((cross OR across OR between) AND boundar* AND (learn* OR collaboration)) OR boundary role OR liaison OR ‘go-between’ OR intermediator OR navigator OR ‘linkage agent’ OR ‘boundary worker’ OR ((linking OR connecting OR integrating) AND boundaries) OR ‘role-model’ OR ‘disseminated learning’ OR socialization OR ‘informal learning’ OR ‘non-formal learning’ OR translator OR facilitator OR ((diffus* OR transfer* OR connect* OR link* OR exchang* OR disseminat* OR shar* OR facilitate*) AND (knowledge OR information)) OR ((link* OR transfer* OR connect* OR work OR working OR exchang* OR communicat*) AND (practices OR organizations OR settings OR workplaces OR institutes OR agencies)) OR ‘organizational barriers’)  Filters on educational level: only higher education and postsecundary education |

**Figure 2: comprehensive search**

| **Databases** | **Synonyms of “clinician”, “teacher” and “integrating clinical practice and education”** |
| --- | --- |
| Scopus | (TITLE-ABS-KEY("clinician teacher*" OR "clinician educator*" OR "clinical teacher*" OR "clinical educator*" OR "practitioner teacher*") OR (TITLE-ABS-KEY(Clinician* OR physician* OR doctor* OR "medical practitioner*" ) AND TITLE-ABS-KEY(Teach OR teaches OR teaching OR teacher* OR educate* OR educating OR educator* OR Instruct* OR Educationalist* OR lecturer*)) OR (TITLE-ABS-KEY(Classroom* OR "pre-clinical" OR bachelor OR "academic half days" OR "academic weeks") AND TITLE-ABS-KEY(Clinician* OR physician* OR doctor* OR "medical practitioner*")) ) AND (TITLE-ABS-KEY(Broker* OR (boundar* W/5 spanner*) OR (boundar* W/5 crosser*) OR gatekeeper* OR brokering OR (boundar* W/5 crossing) OR (boundar* W/5 spanning) OR (((cross OR across OR between) W/5 boundar*) AND (learn* OR collaborat*)) OR (boundar* AND ((cross OR across OR between OR different) W/5 (practices OR organizations OR settings OR workplaces OR institutes OR agencies))) OR "boundary role*" ) OR TITLE-ABS-KEY ( liaison* OR "go between*" OR intermediator* OR navigator* OR "linkage agent*" OR "boundary worker*" OR ( ( linking OR connecting OR integrating ) W/10 boundaries ) OR "role model*" OR "disseminated learning" ) OR ( TITLE-ABS-KEY ( building W/10 bridge* ) AND SUBJAREA ( medi OR nurs OR vete OR dent OR heal OR mult ) ) OR TITLE-ABS-KEY(Socialization OR "informal learning" OR "non-formal learning" OR translator* OR facilitator* OR ((diffuse* OR transfer* OR connect* OR link* OR exchang* OR disseminat* OR shar* OR facilitat*) W/3 (knowledge OR information)) OR ((link* OR transfer* OR connect* OR work OR working OR exchang* OR communicat*) W/5 (practices OR organizations OR settings OR workplaces OR institutes OR agencies)) OR (hybridization W/5 "institutional logics") OR "organizational barrier*")) |
| Pubmed | ((((("clinician educator*"[Title/Abstract]) OR ("clinical teacher*"[Title/Abstract])) OR ("clinical educator*"[Title/Abstract])) OR ("practitioner-teacher*"[Title/Abstract])) OR ("clinician teacher*"[Title/Abstract]) OR ( (((Clinician*[Title/Abstract]) OR (Physician*[Title/Abstract])) OR (Doctor*[Title/Abstract])) OR (“Medical practitioner*”[Title/Abstract]) AND ((((((((((teach[Title/Abstract]) OR (teaches[Title/Abstract])) OR (teaching[Title/Abstract])) OR (teacher*[Title/Abstract])) OR (educate[Title/Abstract])) OR (educates[Title/Abstract])) OR (educating[Title/Abstract])) OR (educator*[Title/Abstract])) OR (instruct*[Title/Abstract])) OR (educationalist*[Title/Abstract])) OR (lecturer*[Title/Abstract]) )OR ( ((((Clinician*[Title/Abstract]) OR (Physician*[Title/Abstract])) OR (Doctor*[Title/Abstract])) OR (“Medical practitioner*”[Title/Abstract]) AND (((((Classroom*[Title/Abstract]) OR ("Pre-clinical"[Title/Abstract]))) OR (Bachelor[Title/Abstract])) OR (“Academic half days”[Title/Abstract])) OR (“academic weeks”[Title/Abstract])) ))AND (( (((((((((((("Dual role*"[Title/Abstract]) OR ("Hybrid role*"[Title/Abstract])) OR ("Double role*"[Title/Abstract])) OR ("Dual function*"[Title/Abstract])) OR ("Hybrid function*"[Title/Abstract])) OR ("Double function*"[Title/Abstract])) OR ("Dual profession*"[Title/Abstract])) OR ("Hybrid profession*"[Title/Abstract])) OR ("Double profession*"[Title/Abstract])) OR ("Two roles"[Title/Abstract])) OR ("Two functions"[Title/Abstract])) OR ("Two professions"[Title/Abstract])) OR ("blended professional*"[Title/Abstract]) ) OR ( (((((((Broker*[Title/Abstract]) OR (boundary spanner[Title/Abstract])) OR (boundary crosser[Title/Abstract])) OR (gatekeeper*[Title/Abstract])) OR (brokering[Title/Abstract])) OR (boundary crossing[Title/Abstract])) OR (boundary spanning[Title/Abstract])) OR ("boundary role*"[Title/Abstract]) OR ((learn*[Title/Abstract]) OR (collaboration*[Title/Abstract]) AND (("cross boundar*"[Title/Abstract]) OR ("across boundar*"[Title/Abstract])) OR ("between boundar*"[Title/Abstract])) OR (((((practices[Title/Abstract]) OR (organisations[Title/Abstract])) OR (settings[Title/Abstract])) OR (workplaces[Title/Abstract])) OR (institutes[Title/Abstract])) OR (agencies[Title/Abstract]) AND (((across[Title/Abstract]) OR (cross[Title/Abstract])) OR (between[Title/Abstract])) OR (different[Title/Abstract]) AND (boundar*[Title/Abstract]) ) OR ( (((((((Liaison*[Title/Abstract]) OR ("Go between*"[Title/Abstract])) OR (Navigator*[Title/Abstract])) OR (Intermediator*[Title/Abstract])) OR ("Linkage agent*"[Title/Abstract])) OR ("Boundary worker*"[Title/Abstract])) OR ("Role-model*"[Title/Abstract])) OR ("Disseminated learning"[Title/Abstract]) OR ((((linking[Title/Abstract]) OR (connecting[Title/Abstract])) OR (integrating[Title/Abstract])) AND (boundaries[Title/Abstract])) OR ((building[Title/Abstract]) AND (bridge*[Title/Abstract])) ) ) |
| EmBase | ( ( 'clinician-teacher*':ti,ab,kw OR 'clinician-educator*':ti,ab,kw OR 'clinical-teacher*':ti,ab,kw OR 'clinical-educator*':ti,ab,kw ) OR ( (clinician:ti,ab,kw OR physician:ti,ab,kw OR doctor*:ti,ab,kw OR 'medical practitioner*':ti,ab,kw) AND (teaching:ti,ab,kw OR teach:ti,ab,kw OR teaches:ti,ab,kw OR teacher:ti,ab,kw OR teachers:ti,ab,kw OR educate:ti,ab,kw OR educates:ti,ab,kw OR educating:ti,ab,kw OR educator*:ti,ab,kw OR instruct*:ti,ab,kw OR educationalist*:ti,ab,kw OR lecturer*:ti,ab,kw) ) OR ((clinician:ti,ab,kw OR physician:ti,ab,kw OR doctor*:ti,ab,kw OR 'medical practitioner*':ti,ab,kw) AND (classroom:ti,ab,kw OR 'pre-clinical':ti,ab,kw OR 'undergraduate education':ti,ab,kw OR 'academic half days':ti,ab,kw OR 'academic weeks':ti,ab,kw))) AND (( broker*:ti,ab,kw OR ((boundary NEAR/5 spanner*):ti,ab,kw) OR ((boundary NEAR/5 crosser*):ti,ab,kw) OR ((boundary NEAR/5 spanning):ti,ab,kw) OR ((boundary NEAR/5 crossing):ti,ab,kw) OR gatekeeper*:ti,ab,kw OR brokering:ti,ab,kw OR 'boundary role':ti,ab,kw OR ((((cross OR across OR between) NEAR/5 boundar*):ti,ab,kw) AND (learn*:ti,ab,kw OR collaboration*:ti,ab,kw)) OR (boundar*:ti,ab,kw AND (((across OR cross OR between OR different) NEAR/5 (practices OR organisations OR settings OR workplaces OR institutes OR agencies)):ti,ab,kw))) OR ( liaison*:ti,ab,kw OR 'go between*':ti,ab,kw OR intermediator*:ti,ab,kw OR navigator*:ti,ab,kw OR 'linkage agent*':ti,ab,kw OR 'boundary worker*':ti,ab,kw OR (((linking OR connecting OR integrating) NEAR/10 boundaries):ti,ab,kw) OR ((building NEAR/10 bridge*):ti,ab,kw) OR 'role-model*':ti,ab,kw OR 'disseminated learning':ti,ab,kw ) OR ( socialization:ti,ab,kw OR 'informal learning':ti,ab,kw OR 'non-formal learning':ti,ab,kw OR translator*:ti,ab,kw OR facilitator*:ti,ab,kw OR ((hybridisation NEAR/5 'institutional logics'):ti,ab,kw) OR 'organizational barriers':ti,ab,kw OR (((diffus* OR transfer* OR connect* OR link* OR exchange* OR exchang* OR disseminat* OR shar* OR facilitate*) NEAR/3 (knowledge OR information)):ti,ab,kw) OR (((link* OR transfer* OR connect* OR work OR working OR exchang* OR communicat*) NEAR/5 (practices OR organizations OR settings OR workplaces OR institutes OR agencies)):ti,ab,kw) )) |
| ERIC | (clinician OR physician OR 'medical practitioner' OR doctor) AND ( (‘clinician educator’ OR ‘clinical educator’ OR ‘clinician teacher’ OR ‘clinical teacher’) OR ((Clinician OR physician OR doctor OR ‘medical practitioner’) AND (teach OR educat OR instructor OR lecturer) ) OR ( (Clinician OR physician OR doctor OR ‘medical practitioner’) AND (classroom OR ‘pre-clinical’ OR bachelor OR ‘academic half days’ OR ‘academic weeks’) )) AND (Broker OR ‘boundary span*’ OR ‘boundary cros*’ OR gatekeeper OR brokering OR ((cross OR across OR between) AND boundar* AND (learn* OR collaboration)) OR boundary role OR liaison OR ‘go-between’ OR intermediator OR navigator OR ‘linkage agent’ OR ‘boundary worker’ OR ((linking OR connecting OR integrating) AND boundaries) OR ‘role-model’ OR ‘disseminated learning’ OR socialization OR ‘informal learning’ OR ‘non-formal learning’ OR translator OR facilitator OR ((diffus* OR transfer* OR connect* OR link* OR exchang* OR disseminat* OR shar* OR facilitate*) AND (knowledge OR information)) OR ((link* OR transfer* OR connect* OR work OR working OR exchang* OR communicat*) AND (practices OR organizations OR settings OR workplaces OR institutes OR agencies)) OR ‘organizational barriers’)  Filters on educational level: only higher education and postsecundary education. |
