# Supplementary material for: Fostering the clinician as teacher: A realist review
Source: Med Educ. 2024 Jul 21;59(2):151–63. doi: 10.1111/medu.15476 (PMC11708814; doi:10.1111/medu.15476)
Supplement: Supplementary file 2 — Appendix B. List of all 66 included articles 1 , 2 , 3 , 4 , 5 , 6 , 7 , 8 , 9 , 10 , 11 , 12 , 13 , 14 , 15 , 16 , 17 , 18 , 19 , 20 , 21 , 22 , 23 , 24 , 25 , 26 , 27 , 28 , 29 , 30 , 31 , 32 , 33 , 34 , 35 , 36 , 37 , 38 , 39 , 40 , 41 , 42 , 43 , 44 , 45 , 46 , 47 , 48 , 49 , 50 , 51 , 52 , 53 , 54 , 55 , 56 , 57 , 58 , 59 , 60 , 61 , 62 , 63 , 64 , 65 , 66 [file MEDU-59-151-s002.docx]

**Appendix B – list of all 66 included articles**^1-66^

1. Kumar K, Greenhill J. Factors shaping how clinical educators use their educational knowledge and skills in the clinical workplace: A qualitative study. BMC Medical Education. 2016;16(1).

2. Møller JE, Kjaer LB, Helledie E, Nielsen LF, Malling BV. Transfer of communication teaching skills from university to the clinical workplace – does it happen? A mixed methods study. BMC Medical Education. 2021;21(1).

3. Chen HC, Fogh S, Kobashi B, Teherani A, ten Cate O, O’Sullivan P. An interview study of how clinical teachers develop skills to attend to different level learners. Medical Teacher. 2016;38(6):578-84.

4. Elmberger A, Blitz J, Björck E, Nieminen J, Bolander Laksov K. Faculty development participants' experiences of working with change in clinical settings. Med Educ. 2023;57(7):679-88.

5. Regan L, Hopson LR, Gisondi MA, Branzetti J. Creating a better learning environment: a qualitative study uncovering the experiences of Master Adaptive Learners in residency. BMC Medical Education. 2022;22(1).

6. Mann KV, Bruce Holmes D, Hayes VM, Burge FI, Viscount PW. Community family medicine teachers' perceptions of their teaching role. Medical Education. 2001;35(3):278-85.

7. Egnew TR, Wilson HJ. Role modeling the doctor-patient relationship in the clinical curriculum. Family Medicine. 2011;43(2):99-105.

8. Kisiel JB, Bundrick JB, Beckman TJ. Resident physicians' perspectives on effective outpatient teaching: A qualitative study. Advances in Health Sciences Education. 2010;15(3):357-68.

9. Santivasi WL, Nordhues HC, Hafferty FW, Vaa Stelling BE, Ratelle JT, Beckman TJ, Sawatsky AP. Reframing professional identity through navigating tensions during residency: A qualitative study. Perspect Med Educ. 2022;11(2):93-100.

10. Houchens N, Harrod M, Fowler KE, Moody S, Saint S. How Exemplary Inpatient Teaching Physicians Foster Clinical Reasoning. The American Journal of Medicine. 2017;130(9):1113.e1-.e8.

11. Yedidia MJ, Schwartz MD, Hirschkorn C, Lipkin Jr M. Learners as teachers - The conflicting roles of medical residents. Journal of General Internal Medicine. 1995;10(11):615-23.

12. Rowe M, Frantz J, Bozalek V. Beyond knowledge and skills: the use of a Delphi study to develop a technology-mediated teaching strategy. BMC Medical Education. 2013;13(1):51.

13. Knight LV, Bligh J. Physicians' perceptions of clinical teaching: A qualitative analysis in the context of change. Advances in Health Sciences Education. 2006;11(3):221-34.

14. Lemaire JB, Miller EN, Polachek AJ, Wong H. Stakeholder Groups’ Unique Perspectives About the Attending Physician Preceptor Role: A Qualitative Study. Journal of General Internal Medicine. 2019;34(7):1158-66.

15. Gonzalo JD, Heist BS, Duffy BL, Dyrbye L, Fagan MJ, Ferenchick G, et al. Content and timing of feedback and reflection: A multi-center qualitative study of experienced bedside teachers. BMC Medical Education. 2014;14(1).

16. Hudak NM, Melcher B, De Oliveira JS. Preceptors’ perceptions of interprofessional practice, student interactions, and strategies for interprofessional education in clinical settings. Journal of Physician Assistant Education. 2017;28(4):214-7.

17. Sternszus R, Boudreau JD, Cruess RL, Cruess SR, MacDonald ME, Steinert Y. Clinical Teachers' Perceptions of Their Role in Professional Identity Formation. Academic Medicine. 2020:1594-9.

18. Wright SM, Carrese JA. Excellence in role modelling: Insight and perspectives from the pros. CMAJ. 2002;167(6):638-43.

19. Barnhoorn PC, Nierkens V, Numans ME, Steinert Y, van Mook WNKA. “What kind of doctor do you want to become?”: Clinical supervisors’ perceptions of their roles in the professional identity formation of General Practice residents. Medical Teacher. 2023;45(5):485-91.

20. Mazor KM, Fischer MA, Haley HL, Hatem D, Quirk ME. Teaching and medical errors: primary care preceptors' views. Med Educ. 2005;39(10):982-90.

21. Butani L, Paterniti DA, Tancredi DJ, Li STT. Attributes of residents as teachers and role models-A mixed methods study of stakeholders. Medical Teacher. 2013;35(4):e1052-e9.

22. Maker VK, Curtis KD, Donnelly MB. Are you a surgical role model? Current Surgery. 2004;61(1):111-5.

23. Wray N, McCall L. "They Don't Know Much about Us": Educational Reform Impacts on Students' Learning in the Clinical Environment. 2009;14(5):665-76.

24. Martens MJC, Duvivier RJ, Van Dalen J, Verwijnen GM, Scherpbier AJJA, Van Der Vleuten CPM. Student views on the effective teaching of physical examination skills: A qualitative study. Medical Education. 2009;43(2):184-91.

25. Ramani S, Orlander JD. Human Dimensions in Bedside Teaching: Focus Group Discussions of Teachers and Learners. Teaching and Learning in Medicine. 2013;25(4):312-8.

26. Lim DW, White JS. How do surgery students use written language to say what they see? A framework to understand medical students' written evaluations of their teachers. Academic Medicine. 2015;90(11 Association of American Medical Colleges Medical Education Meeting):S98-S106.

27. Habboush Y, Stoner A, Torres C, Beidas S. Implementing a clinical-educator curriculum to enrich internal medicine residents' teaching capacity. BMC Medical Education. 2019;19(1).

28. Brown J, Reid H, Dornan T, Nestel D. Becoming a clinician: Trainee identity formation within the general practice supervisory relationship. Medical Education. 2020;54(11):993-1005.

29. Gran SF, Brænd AM, Lindbæk M, Frich JC. General practitioners’ and students’ experiences with feedback during a six-week clerkship in general practice: a qualitative study. Scandinavian Journal of Primary Health Care. 2016;34(2):172-9.

30. Scholz A, Gehres V, Schrimpf A, Bleckwenn M, Deutsch T, Geier AK. Long-term mentoring relationships in undergraduate longitudinal general practice tracks - a qualitative study on the perspective of students and general practitioners. Med Educ Online. 2023;28(1):2149252.

31. Harms S, Bogie BJM, Lizius A, Saperson K, Jack SM, McConnell MM. From good to great: learners' perceptions of the qualities of effective medical teachers and clinical supervisors in psychiatry. Can Med Educ J. 2019;10(3):e17-e26.

32. DeWitt DE, Migeon M, LeBlond R, Carline JD, Francis L, Irby DM. Insights from outstanding rural internal medicine residency rotations at the University of Washington. Academic Medicine. 2001;76(3):273-81.

33. Althouse LA, Stritter FT, Steiner BD. Attitudes and Approaches of Influential Role Models in Clinical Education. Advances in Health Sciences Education. 1999;4(2):111-22.

34. Sandhu G, Rich JV, Magas C, Walker GR. A Diverging View of Role Modeling in Medical Education. 2015;6(1).

35. Wright SM, Carrese JA. Serving as a physician role model for a diverse population of medical learners. Academic Medicine. 2003;78(6):623-8.

36. Stenfors-Hayes T, Berg M, Scott I, Bates J. Common concepts in separate domains? Family physicians' ways of understanding teaching patients and trainees, a qualitative study. BMC Medical Education. 2015;15(1).

37. Noble C, Hilder J, Billett S, Teodorczuk A, Ajjawi R. Supervisory knowing in practice across medical specialities. Advances in Health Sciences Education. 2023.

38. Giroldi E, Veldhuijzen W, Geelen K, Muris J, Bareman F, Bueving H, et al. Developing skilled doctor-patient communication in the workplace: a qualitative study of the experiences of trainees and clinical supervisors. Adv Health Sci Educ Theory Pract. 2017;22(5):1263-78.

39. Sagasser MH, Kramer AW, van Weel C, van der Vleuten CP. GP supervisors' experience in supporting self-regulated learning: a balancing act. Adv Health Sci Educ Theory Pract. 2015;20(3):727-44.

40. Goldie J, Dowie A, Goldie A, Cotton P, Morrison J. What makes a good clinical student and teacher? An exploratory study Approaches to teaching and learning. BMC Medical Education. 2015;15(1).

41. Pearson D, Lucas B. What are the key elements of a primary care teaching practice? Education for Primary Care. 2011;22(3):159-65.

42. Weissmann PF, Branch WT, Gracey CF, Haidet P, Frankel RM. Role modeling humanistic behavior: Learning bedside manner from the experts. Academic Medicine. 2006;81(7):661-7.

43. Muller-Juge V, Pereira Miozzari AC, Rieder A, Hasselgård-Rowe J, Sommer J, Audétat MC. A medical student in private practice for a 1-month clerkship: a qualitative exploration of the challenges for primary care clinical teachers. Adv Med Educ Pract. 2018;9:17-26.

44. Stenfors-Hayes T, Hult H, Dahlgren LO. What does it mean to be a good teacher and clinical supervisor in medical education? Advances in Health Sciences Education. 2011;16(2):197-210.

45. Shapiro J. How do physicians teach empathy in the primary care setting? Academic Medicine. 2002;77(4):323-8.

46. Côté L, Laughrea PA. Preceptors' understanding and use of role modeling to develop the CanMEDS competencies in residents. Academic Medicine. 2014;89(6):934-9.

47. Smith CC, Newman LR, Huang GC. Those Who Teach, Can Do: Characterizing the Relationship Between Teaching and Clinical Skills in a Residency Program. Journal of graduate medical education. 2018;10(4):459-63.

48. Bartle E, Thistlethwaite J. Becoming a medical educator: Motivation, socialisation and navigation. BMC Medical Education. 2014;14(1).

49. Chen LYC, Hubinette MM. Exploring the role of classroom-based learning in professional identity formation of family practice residents using the experiences, trajectories, and reifications framework. Medical Teacher. 2017;39(8):876-82.

50. Hu WCY, Thistlethwaite JE, Weller J, Gallego G, Monteith J, McColl GJ. ‘It was serendipity’: a qualitative study of academic careers in medical education. Medical Education. 2015;49(11):1124-36.

51. Hendry RG, Kawai GK, Moody WE, Sheppard JE, Smith LCR, Richardson M, et al. Consultant attitudes to undertaking undergraduate teaching duties: Perspectives from hospitals serving a large medical school. Medical Education. 2005;39(11):1129-39.

52. Minor S, Huffman M, Lewis P, Kost A, Prunuske J. Community preceptor perspectives on recruitment and retention: The CoPPRR study. Family Medicine. 2019;51(5):389-98.

53. Koppula S, Brown Dr JB, Jordan Dr JM. Teaching primary care obstetrics, Insights and recruitment recommendations from family physicians. Canadian Family Physician. 2014;60(3):e180-e6.

54. Murakami M, Kawabata H, Maezawa M. What primary care physician teachers need to sustain community based education in Japan. Asia Pacific Family Medicine. 2014;13(1).

55. Cantillon P, D'Eath M, De Grave W, Dornan T. How Do Clinicians Become Teachers? A Communities of Practice Perspective. 1008;21(5):991-1008.

56. Cochran Ward E, Kwan J, Garlan K, Bassett E, Klein L. 'To teach or not to teach?' Factors that motivate and constrain Australian emergency medicine physicians to teach medical students. EMA - Emergency Medicine Australasia. 2013;25(4):353-8.

57. Stark P. Teaching and learning in the clinical setting: A qualitative study of the perceptions of students and teachers. Medical Education. 2003;37(11):975-82.

58. Beckwith H, Kingsbury M, Horsburgh J. Why do people choose nephrology? Identifying positive motivators to aid recruitment and retention. Clin Kidney J. 2018;11(5):599-604.

59. Dixon, Lam, Lam. Does a brief clerkship change Hong Kong medical students’ ideas about general practice? Medical Education. 2000;34(5):339-47.

60. Barone MA, Dudas RA, Stewart RW, McMillan JA, Dover GJ, Serwint JR. Improving teaching on an inpatient pediatrics service: A retrospective analysis of a program change. BMC Medical Education. 2012;12(1).

61. Sheu L, Charondo L, O’Sullivan P. Faculty motivations for leading clinical clerkship electives: A qualitative study. Medical Teacher. 2022;44:1-7.

62. Bank L, Jippes M, van Rossum TR, den Rooyen C, Scherpbier AJJA, Scheele F. How clinical teaching teams deal with educational change: ‘we just do it’. BMC Medical Education. 2019;19(1):377.

63. Fokkema JP, Westerman M, Teunissen PW, van der Lee N, Scherpbier AJ, van der Vleuten CP, et al. How lead consultants approach educational change in postgraduate medical education. Med Educ. 2012;46(4):390-8.

64. Elmberger A, Björck E, Nieminen J, Liljedahl M, Bolander Laksov K. Collaborative knotworking – transforming clinical teaching practice through faculty development. BMC Medical Education. 2020;20(1).

65. Ahluwalia S, Spicer J, Patel A, Cunningham B, Gill D. Understanding the relationship between GP training and improved patient care - a qualitative study of GP educators. Educ Prim Care. 2020;31(3):145-52.

66. Elmberger A, Björck E, Liljedahl M, Nieminen J, Bolander Laksov K. Contradictions in Clinical Teachers' Engagement in Educational Development: An Activity Theory Analysis. 2019;24(1):125-40.
