# Supplementary material for: Fostering the clinician as teacher: A realist review
Source: Med Educ. 2024 Jul 21;59(2):151–63. doi: 10.1111/medu.15476 (PMC11708814; doi:10.1111/medu.15476)
Supplement: Supplementary file 3 — Appendix C. Supportive quotes for CMO constructions [file MEDU-59-151-s003.docx]

**Appendix C – Supportive quotes for CMO constructions**

| **Nb** | **CMO** | **Supportive quote** |
| --- | --- | --- |
| CMO1 | In a context where the CTs educational role and profile is formally recognized and supported by colleagues and management (context), the CT shared educational tactics and skills with colleague physicians (resource mechanism), who ascribed a high level of credibility to this information (reasoning mechanism) and hence implemented educational tactics in their own teaching with students (outcome). | *Colleague physician: ‘I don’t think we make use of it, and I think that’s too bad […] because they know stuff that we, their colleagues, could learn from. They didn’t need to keep quiet about that.* - Art 3 |
| CMO2 | In a context where colleague physicians physicians (with less educational experience than the CT) display inadequate educational expertise when working with students or colleagues (context), the CT offered relevant practical teaching recommendations, specific and appropriate to the clinical context (resource mechanism) that facilitated an understanding of their relevance and a positive disposition towards the recommendations offered (reasoning mechanism). Subsequently colleague physicians applied some teaching techniques in practice (outcome). | *“I used it at the psychiatric department where I*  *taught feedback at a meeting. And people were*  *happy about it. It was really only that you had an*  *optional theme you could choose and I chose collegial supervision as the theme and taught a bit about communication and giving feedback to colleagues. I chose that because I had witnessed some situations that weren’t ideal. So, I thought there was something my colleagues could learn.* *A lot of them said afterwards that they had used it and it was really good. However, there wasn’t any follow up*. Art 13 |
| CMO3 | In an educational system, either within clinical or classroom setting, with sufficient allocated time and equipped space for CTs educational responsibilities (context), the CT admitted his own limitations and uncertainties with regard to patient care to the students (resource mechanism). This made students appreciate the transparency and humanity shown and made them feel legitimized to express their limitations in patient care as well (reasoning mechanism). This resulted in a safe and stimulating learning environment (outcome). | *‘One of my first attendings in medical school told me, be kind to yourself, and understand that medicine is sort of a journey. . . If you still aren’t learning things when you’re 70, then you’re doing something wrong. . . And I think because of that, I’ve been able to accept that I’m in for a long ride and not feel rushed to learn everything rapidly, and if I can just learn a couple of new things a day, then I’m on the right track. – art 52* |
| CMO4 | In an educational system, either within clinical or classroom setting, with sufficient allocated time and equipped space for CTs educational responsibilities (context), the CT provided students with learning opportunities and autonomy appropriate to the students’ level of competence (resource mechanism), making the students appreciate and feel empowered by the well-aligned tasks (reasoning mechanism), resulting in the growth of the students’ clinical competence (outcome). | *Residents accommodate the heavy job demands placed on them as team leaders by assigning teaching a secondary priority. Such adaptations make the work more manageable but, as one resident acknowledged, have their shortcomings for interns: As an intern, that's a drag. All of these interesting decisions are being made, and you're just being told to bring the tubes to the fourth floor.* |
| CMO5 | In an educational system, either within clinical or classroom setting, with sufficient allocated time and equipped space for CTs educational responsibilities (context), the CT explicitly explained the clinical reasoning behind his actions to the students (resource mechanism); this transparency fostered a deeper understanding of the observed in students (reasoning mechanism), and therefore expedited their learning (outcome). | *Student: The CT [who had allocated time for his teaching role] always explains what she is thinking and why she makes certain decisions. This really expedites learning because it does not leave students to guess and try to make sense of everything on their own*. – art 41 |
| CMO6 | In a context where the CTs’ clinical role is recognized and valued by students in the classroom (outcome), the CT taught students by means of an active role model function and thereby showed students they are still actively working as a physician (resource mechanism), the students ascribed a high level of credibility and legitimacy towards the CT, leading to the belief the information is useful (reasoning mechanism), which resulted in enhancement of students’ competency (outcome). | *CT “Probably from my own experience, if somebody was trying to teach me a skill or what have you and they weren’t working as a doctor at the time, I’d probably think, I’m not sure that you know what you’re talking about because you’re just sitting in an office all day. So seeing them as well in the clinical context is important to reinforce that you’re still working and what you’re saying is probably useful.’ –* art 99 |
| CMO7 | In a context where the CT is viewed as competent and credible teacher by faculty members in the medical school (context), the CT voiced concerns about the mismatches between clinical practice and the curriculum (resource mechanism), which faculty members valued as this feedback came from clinical practice (reasoning mechanism), therefore resulting in curriculum changes (outcome). | Authors*: Preceptors at three outstanding rural residency sites were interviewed about their experiences, teaching strategies and opinions about curriculum. […] The suggestions of [these] preceptors should be used to develop and disseminate a curriculum that will better prepare residents for rural practice.* – art 71  (neg) *‘Some consultants made clear their perception that their concerns about curricular changes had not been listened to and that the medical school expected them to deliver undergraduate teaching without complaint and without reward. For some, this resulted in disappointment at that lack of any form of thanks or formal recognition for their teaching-related work.’* – art 73 |
| CMO8 | In a context where students have unfamiliarity or misconceptions concerning a specialty with shortages of physicians (context), the CT took the time and effort to show students the entire spectrum of their specialty in order to give them a realistic image of the profession, including how to manage work-life balance (resource mechanism), the student in turn manages to visualize themselves in this specialty (reasoning mechanism), and as a result displayed more interest in the specialty and considered this specialty as a future career choice. (outcome) | *The preceptors of our study were excited about sharing the full spectrum of their lives with residents including recreational activities, lifestyles, their sense of community service and professionalism and their decision to practice rural medicine. […] Of the ten residents who completed their rotations, all felt the experience had increased their confidence to practice in a rural setting. The residents agreed that the rotation had made them more likely to choose rural practice.*  – art 71 |
| CMO9 | In a context where the clinical team displays resistance to educational innovation (context), the CT negotiated goals and adjusted plans for the implementation of educational innovations (resource mechanism) in a manner that resulted in feelings of being in control and shared responsibility on the part of the clinical team (reasoning mechanism) leading to the implementation of educational changes. (outcome). | *[colleagues] pointed out that changing one’s own behaviour is really difficult as it requires adjustment of personal routines. Time constraints and demands in patient care could subvert change efforts and make team members easily slip back into old habits. Also, the feeling of not being involved made [colleagues] the feeling that they were not in control over an unreasonable change*. – art E1 |
| CMO10 | In a context where CTs are valued for their clinical role as part of the clinical team (context), the CT had a mandate to contribute suggestions and advice towards educational innovation for the clinical team (resource mechanism); the suggestions were perceived as trustworthy by colleagues involved in the change (reasoning mechanism), which led to changes in educational practices (outcome). | *The participants noted the importance of being perceived as in-group in the workplace as this contributed to a mandate to change and discussed that this could be achieved by having team members with the same professional belonging*– art 8 |
| CMO11 | In a context where the CTs educational role and profile is formally recognized and where the CT feels supported by management and colleagues (context), the CT was able to challenge educational thinking and practices in the organization through the use of relevant and innovative evidence (resource mechanism), this led to credibility bestowed by the clinical team on the CT (reasoning mechanism), leading to design and implementation of educational innovation (outcome). | *Workplace managers […] also noted some CTs [with formal educational roles] were able to extend or challenge current educational thinking and practice within the organization in terms of ‘working with students in ways not thought of before]. – art 3* |
